# Young Children Consider Merit when Sharing Resources with Others

**DOI:** 10.1371/journal.pone.0043979

**Published:** 2012-08-29

**Authors:** Patricia Kanngiesser, Felix Warneken

**Affiliations:** 1 School of Experimental Psychology, University of Bristol, Bristol, United Kingdom; 2 Department of Psychology, Harvard University, Cambridge, Massachusetts, United States of America; Boston College, United States of America

## Abstract

Merit is a key principle of fairness: rewards should be distributed according to how much someone contributed to a task. Previous research suggests that children have an early ability to take merit into account in third-party situations but that merit-based sharing in first-party contexts does not emerge until school-age. Here we provide evidence that three- and five-year-old children already use merit to share resources with others, even when sharing is costly for the child. In Study 1, a child and a puppet-partner collected coins that were later exchanged for rewards. We varied the work-contribution of both partners by manipulating how many coins each partner collected. Children kept fewer stickers in trials in which they had contributed less than in trials in which they had contributed more than the partner, showing that they took merit into account. Few children, however, gave away more than half of the stickers when the partner had worked more. Study 2 confirmed that children related their own work-contribution to their partner’s, rather than simply focusing on their own contribution. Taken together, these studies show that merit-based sharing is apparent in young children; however it remains constrained by a self-serving bias.

## Introduction

Merit is a key principle for fair resource distribution: rewards should reflect how much someone contributed to a task. This principle has been discussed in Western philosophical traditions on distributive justice [1] and is known to guide resource sharing in adults [2]. However, current consensus is that merit-based sharing emerges later in development, not before school-age or even adolescence [3], [4]. Children are thought to go through three major developmental stages: young children are purely selfish, older children follow a strict equality rule (everyone gets the same, irrespective of individual contributions), and school-aged children begin to take individual contributions into account (“merit” or “equity”, e.g., [5]). According to this account, merit-based sharing is not a fundamental principle characterizing early sharing behaviors. Rather, it might require complex reasoning skills and extensive social practice (potentially including explicit teaching) for young children to overcome their selfish inclinations and to attend to the deservingness of others.

Evidence for this traditional developmental model comes from studies using *hypothetical* scenarios of resource allocation among third parties (e.g., [6]) or from studies in which sharing is *costly* for the child (e.g., [7]). Both types of studies find that pre-school children do not spontaneously share according to merit [4]. Starting at around six to seven years of age, children begin to consistently share more of their resources with someone who has worked more (“ordinal equity”), but only teenagers will share rewards by matching effort and reward proportionally (“proportional equity”; [8], [9]).

These studies, however, used methods that might be very challenging for young children, potentially masking their early competence. Specifically, in previous studies, children were partnered with a fictitious other child or had to share large quantities of up to 20 rewards (e.g., [7], [9]). However, when presented with a simple story, 3-year-olds reasoned that a character who finished baking deserved more cookies than a character who got bored and stopped early [10]. Moreover, it was recently found that 20-month-old infants look longer at scenes in which two characters were rewarded equally, even though only one of them performed all the work [11], indicating that infants may already possess implicit expectations about merit-based reward allocations. Although suggestive, these two studies have only measured competence regarding third-person scenarios. It thus remains an open question whether children will act in accordance with this proficiency when sharing resources in first-person situations in which they are potential recipients of the rewards.

In the current study, we used a novel, interactive game, in which children were partnered with an animated puppet and could decide individually how to share a small quantity of rewards. Specifically, each partner played a “fishing game” to retrieve coins. In two trials, we varied work-contribution by manipulating how many coins the child and the puppet retrieved, respectively. At the end of each trial, we asked children to distribute six prizes (stickers) between themselves and their puppet-partner. We evaluated whether children kept more rewards after contributing more work than after contributing less work.

## Experiment 1

### Methods

#### Participants

We tested 18 three-year-olds (*Mean*: 3;6 years, *Range*: 3;1–3;12 years, 9 female) and 18 five-year-olds (*Mean*: 5;6 years, *Range*: 5;0–5;11, 9 female). Two additional 3-year-olds were excluded due to insufficient English skills, and two five-year-olds due to experimenter error. All children were tested individually in a psychological laboratory and were recruited from a database of families from the Greater Boston area. The Harvard University Committee on the Use of Human Subjects in Research approved the ethics of this study. Informed consent, in written form, was obtained from the parents of all children who participated in this study.

#### Procedure

**Figure 1:**
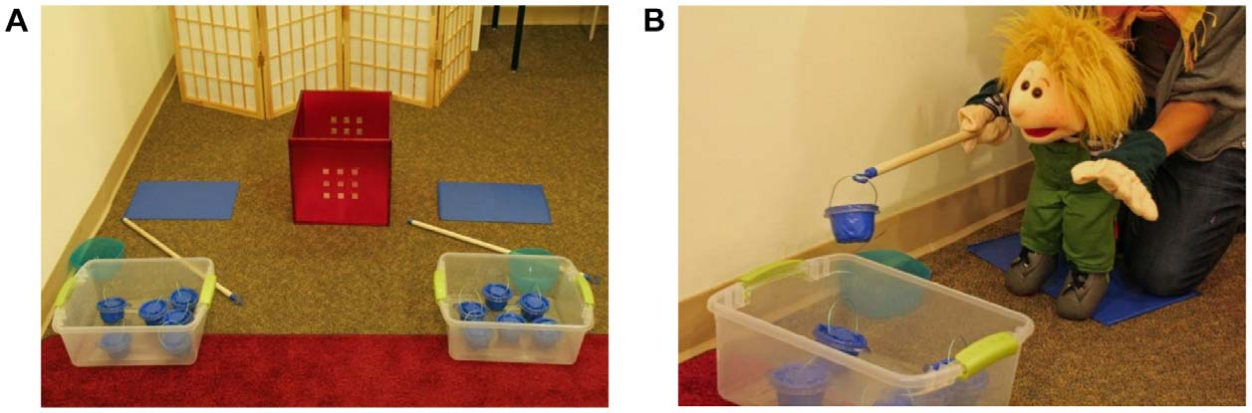
Experimental Set-up. Set-up for the game that children played with a puppet partner (B) to retrieve coins that they were later rewarded for with stickers.

We first familiarized the child with a large hand-puppet of the same gender as the child and introduced the “fishing game.” One experimenter (E1) led the session, while a second experimenter (E2) operated the puppet, never breaking character. We used a puppet as a partner for the children in order to enable us to manipulate the amount of work the partner contributed to the game. In the game, the puppet and the child each sat on the floor in front of a box with six small baskets containing one coin each (see Figure 1). Each player had to collect coins by retrieving the baskets from the respective boxes using a fishing-stick. While sitting about 30 cm away from the box, the players had to reach out with the stick, hook it onto the handle of a basket and lift the basket out of the box. The baskets had tightly fit lids, and they required some effort to open them to retrieve the coins. In order to manipulate the number of coins the puppet and the child collected, the puppeteer’s work-speed was adjusted to that of the child, while E1 started and stopped the game by pretending to use a stopwatch. The experiment consisted of one training trial to introduce the game and two test trials.

In the training trial, E1 stopped the game after the child and the puppet had each retrieved four coins. E1 then counted out loud how many coins each player had retrieved by placing each player’s coins in a separate row on a vertical board. To ensure that the child remembered the number of coins, E1 then turned the board away from the child and puppet and asked the child how many coins each player had collected. Children who did not answer correctly were shown the board again and could indicate the correct number by pointing to the different rows on the board. Next, the child and the puppet each received four stickers and a bag with their initials for storing the stickers. To avoid explicit priming of merit, both partners found the same number of coins during the training trial. Therefore, unequal work occurred for the first time during the test trials.

The two subsequent test trials were identical to the training trial, with the exception that the child either found more or less coins than the puppet. In the *more-work condition* (1 trial), the child found four coins while the puppet only found two, whereas in the *less-work condition* (1 trial), the child found two coins and the puppet found four (order counterbalanced between subjects). At the end of each trial, E1 counted out six stickers and told both players that they could keep some of the stickers. E1 then asked the child whether s/he could help with distributing the stickers, while the puppet left the room to avoid influencing the child. Next, E1 had the child point to the child’s bag and the puppet’s bag, respectively. E1 then instructed the child to put the stickers in the bags while E1 went behind an occluder to ensure privacy. The coin board was visible during the entire distribution task. After the child had distributed all of the stickers (E1 asked after 30 seconds whether the child had finished), E1 re-checked that the child correctly remembered each player’s bag and the number of coins each player had retrieved.

#### Data coding and analysis

All data were coded from video-recordings. 30% of the data were independently coded by a second person to assess inter-rater reliability. We measured how many stickers the child put into the respective bags (κ = 1.0).

We analyzed the data using repeated-measures ANOVAs (with condition as a within-subjects variable and age as a between-subjects variable), Wilcoxon-tests, and exact χ^2^-tests (all tests two-tailed). In addition, we used one-sample t-tests (two-tailed) to examine whether children’s sharing behavior in the two conditions deviated from equal allocations. Children correctly identified the sticker bags and indicated the correct number of coins (two five-year-olds needed more than one repetition after one of the conditions). Two three- and two five-year-olds looked at the coin board in at least one of the conditions while sharing the stickers. Preliminary analysis ruled out effects of gender, *F*_1,32_<.001, *p*>.999, *η*_p_^2^<.001, and condition-order, *F*_1,32_ = 71, *p = *.405, *η*_p_^2^ = 022, on sharing behavior.

### Results and Discussion

**Figure 2:**
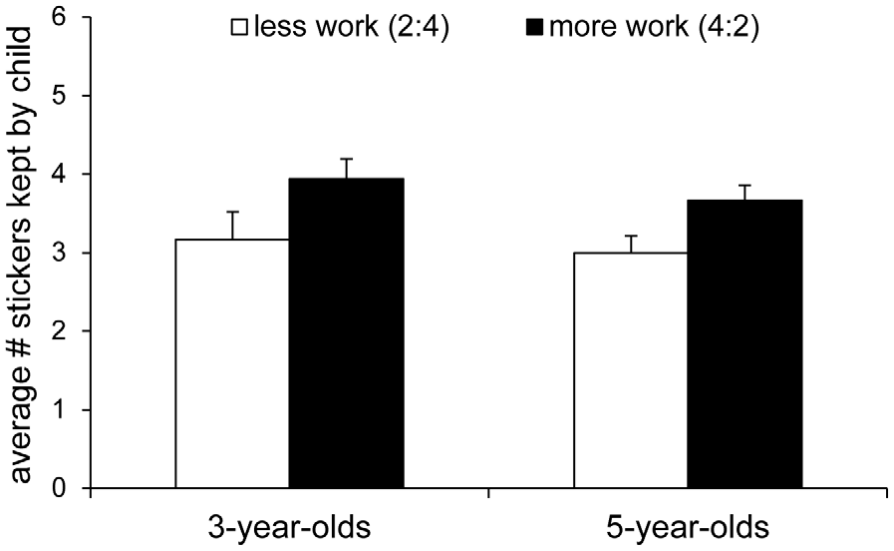
Average amount of stickers that three- and five-year-olds kept for themselves in Experiment 1. Average amount of stickers that three- and five-year-olds kept for themselves in Experiment 1. In the less-work condition (white bars), the child and the puppet retrieved two and four coins, respectively, and in the more-work condition (black bars), the child and the puppet retrieved four and two coins, respectively.

We first examined children’s sharing behavior across the two conditions, finding that three- and five-year-olds kept, on average, significantly more stickers for themselves in the more-work condition than in the less-work condition (see Figure 2), *F*_1,34_ = 16.32, *p*<.001, *η*_p_^2^ = 324. Three- and five-year-olds did not differ significantly in their sharing behavior, *F*_1,34_ = 47, *p = *496, *η*_p_^2^ = 014, nor was there a significant interaction between condition and age, *F*_1,34_ = 10, *p = *758, *η*_p_^2^ = 003. Non-parametric tests confirmed that children kept more stickers in the more-work condition than in the less-work condition, Z = −3.45, p<.001 (17 positive ranks, 3 negative ranks, 16 ties). This finding shows that children as young as three years of age took the different work-contributions into account when sharing rewards with others.

While merit influenced sharing, our results also showed that some children exhibited a self-serving bias. Specifically, across the two conditions, children tended to keep significantly more than half of the stickers for themselves (M = 6.89 out of 12 stickers overall, SD = 1.92), *t*(35) = 2.77, *p = *009, *d* = 0.94. Broken down by condition, children in the more-work condition kept significantly more than half of the stickers for themselves, *t*(35) = 5.08, *p*<.001, *d* = 1.72, while they did not deviate significantly from an equal share in the less-work condition, *t*(35) = 0.41, *p = *686, *d* = 0.14. Thus, even though children were clearly able to consider different work contributions, this tendency was constrained by a self-serving bias.

Next, we looked at individual sharing strategies. Across the two trials, 39% of children kept more than 6 of the 12 stickers for themselves (44% three-year-olds, 33% five-year-olds), 50% of children kept exactly half (39% three-year-olds, 61% five-year-olds), and 11% of children kept less than half (17% three-year-olds, 6% five-year-olds). However, half of the children showed a sensitivity to merit and kept fewer stickers in the less-work than in the more-work condition, with no effect of age (44% three-year-olds, 50% five-year-olds), *χ^2^*(1, *N* = 36) = 0.11, *p*>999. This outcome was due to a number of different sharing strategies, details of which can be found in Table 1. Importantly, 22% of three-year-olds and 28% of five-year-olds kept fewer than three of the six stickers in the less-work condition and more than three stickers in the more-work condition. The rest of the children showed no sensitivity to merit (56% three-year-olds, 50% five-year-olds; *χ^2^*(1, *N* = 36) = 0.11, *p*>999) and either kept more stickers in the less-work condition than in the more-work condition or kept the same number of stickers in both conditions.

**Table 1:** Three- and five-year-olds’ individual sharing strategies in Experiment 1.

	3-year-olds		5-year-olds	
	N	Percent	N	Percent
1 Merit	8	44%	9	50%
*(1a Less/more* a *)*	*(4)*	*(22%)*	*(5)*	*(28%)*
*(1b Less/equal* b *)*	*(3)*	*(17%)*	*(0)*	*(0%)*
*(1c Equal/more* c *)*	*(0)*	*(0%)*	*(3)*	*(17%)*
*(1d More/more* d *)*	*(1)*	*(6%)*	*(1)*	*(6%)*
2 No Merit	10	56%	9	50%
*(2a More to self* e *)*	*(6)*	*(33%)*	*(1)*	*(6%)*
*(2b Equal share* f *)*	*(3)*	*(17%)*	*(6)*	*(33%)*
*(2c Reversal* g *)*	*(1)*	*(6%)*	*(2)*	*(11%)*
Total	18	100%	18	100%

a Keeping less than 3 of 6 stickers in the less-work condition and more than 3 of 6 stickers in the more-work condition.

b Keeping less than 3 of 6 stickers in the less-work condition and sharing stickers equally in the more-work condition.

c Sharing equally in the less-work condition and keeping more than 3 of 6 stickers in the more-work condition.

d Keeping more than 3 of 6 stickers in the less-work condition and even more stickers in the more-work condition.

e Keeping more for themselves with no difference between conditions.

f Always sharing equally.

g Keeping more stickers in the less-work than in the more-work condition.

Taken together, the first set of analyses show that allocations vary as a function of condition, and the second set of analyses show that half of the children were sensitive to merit. Several children even gave more than half of the resources to a partner who had worked more than them.

Despite the differences in sharing behavior between conditions, it remains unclear whether children actually related their own efforts to their partner’s efforts or only paid attention to their own absolute work-effort when sharing rewards (e.g. “I got four coins, hence I get four stickers.”). In fact, Nelson and Dweck [12] found that four-year-olds only orientated their sharing behavior on their own absolute contribution. We thus conducted a second experiment to control for this possibility.

## Experiment 2

In this experiment, the child and the puppet-partner found the same number of coins –either two or four coins each – and the child had to allocate six stickers. If children only focused on their own contribution, ignoring their partner’s contribution, they should keep more stickers in the 4∶4-coin condition than in the 2∶2-coin condition (alike to their allocations in Experiment 1). However, if children evaluated their own contribution relative to their partner’s, they should give equally in both conditions.

### Methods

#### Participants

We tested 18 three-year-olds (*Mean*: 3;5 years, *Range*: 3;2–3;11 years, 9 females) and 18 five-year-olds (*Mean*: 5;4 years, *Range*: 5;0–5;11 years, 9 females) from the same population as Experiment 1. Three additional three-year-olds were excluded because they failed to play the game.

#### Procedure

The procedure was identical to Experiment 1, except that we compared a *2∶2-coin condition* (1 trial), in which each player found two coins, with a *4∶4-coin condition* (1 trial), in which each player found four coins (order counterbalanced between subjects). As in Experiment 1, children were asked to distribute six stickers at the end of each trial.

#### Data coding and analyses

Data coding and analyses were identical to Experiment 1 (agreement between coders: κ = 1.0). Children correctly identified the sticker bags and indicated the correct number of coins before the two test conditions (one 3-year-old failed in one of the conditions). A preliminary analysis showed that the order in which conditions were presented had no effect on sharing behavior, *F*_1,32_ = 49, *p = *488, *η*_p_^2^ = 015.

### Results and Discussion

**Figure 3:**
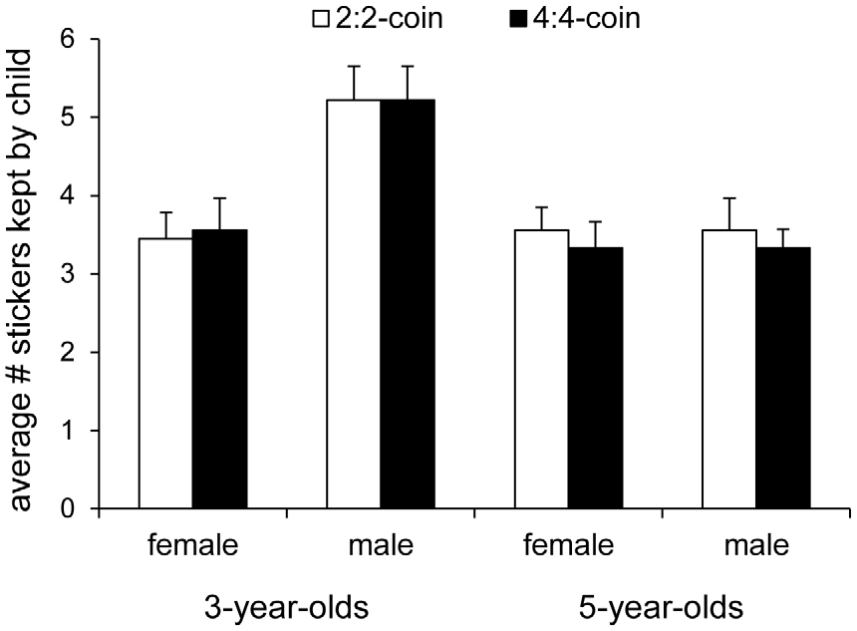
Average amount of stickers that three- and five-year-olds kept for themselves in Experiment 2. Average amount of stickers that three- and five-year-olds kept for themselves in Experiment 2. Data was split by gender due to a significant age×gender interaction. In the 2∶2-coin condition (white bars), the child and the puppet retrieved two coins each, and in the 4∶4-coin condition (black bars), the child and the puppet retrieved two coins each.

We found that children’s sharing behavior did not differ significantly between the 2∶2- and the 4∶4-coin conditions, *F*_1,32_ = 24, *p = *631, *η*_p_^2^ = 007 (see Figure 3). These findings indicate that children’s sharing behavior is not just determined by their own absolute work-effort. Rather, together with the results from Experiment 1, children appear to take into account their own and their partner’s relative contributions when allocating resources.

In contrast to Experiment 1, we found a significant effect of age, *F*_1,32_ = 7.93, *p = *008, *η*_p_^2^ = 199, a significant effect of gender, *F*_1,32_ = 7.00, *p = *013, *η*_p_^2^ = 180, and a significant age×gender interaction, *F*_1,32_ = 7.00, *p = *013, *η*_p_^2^ = 180. Specifically, five-year-old boys and girls did not deviate significantly from an equal share in the 2∶2-coin condition (boys: *t*(8) = 1.35, *p = *214, *d* = 0.96; girls: *t*(8) = 1.89, *p = *095, *d* = 1.34), as well as in the 4∶4-coin condition (boys: *t*(8) = 1.41, *p = *195, *d* = 1.00; girls: *t*(8) = 1.00, *p = *347, *d* = 0.71; see Figure 3). Similarly, three-year-old girls did not deviate significantly from an equal share in the 2∶2-coin condition, *t*(8) = 1.32, *p = *.225, *d* = 0.93, and the 4∶4-coin condition, *t*(8) = 1.35, *p = *214, *d* = 0.96. In contrast, three-year-old boys kept significantly more stickers for themselves in both conditions (*t_s_*(8) = 5.12, *p_s_* = 001, *d_s_* = 3.62). To date, there is mixed evidence regarding gender differences in sharing behavior with some studies finding that young girls are more generous than boys (e.g., [13]) and others not finding any gender differences (e.g., [14]).

## General Discussion

We used a novel experimental paradigm to investigate whether three- and five-year-olds would share rewards with others based on merit. While previous research suggested a late onset of merit-based sharing in first-party contexts, we found that children already possess this propensity by three years of age. Notably, children in our study related their contributions to their partner’s contributions. Young children thus appear to be more flexible and sophisticated in their own sharing behaviour than previously shown. This challenges the traditional notion that merit requires complex reasoning or develops only gradually after extensive social experience during school-age [4]. These findings are consistent with recent studies showing that children expect and approve that a worker should receive more than a non-worker when presented with simplified third party scenarios [10], [11]. Our study, however, shows that children are able to consider different amounts of work-contribution (as opposed to only considering work versus non-work) and, more importantly, use the merit principle not only when judging third parties, but also in first-party situations in which children are potential recipients of rewards.

Previous studies investigating merit-based sharing behaviour have often relied on sharing with anonymous or fictitious partners [4]. In contrast, our sharing task was embedded in a social context in which children directly interacted with a partner. It is conceivable that this context better enabled young children to acknowledge others’ needs and desires and thus could have helped them to acknowledge different work-contributions when sharing actual resources. Similarly, 3-year-olds were recently found to share rewards equally with a peer in concrete situations involving joint collaboration [15], [16]. We extend these findings by showing that young children also consider merit when sharing rewards with others. Furthermore, whereas in the studies by Hamann et al. [15] and Warneken et al. [16], both partners could haggle over the rewards, our study required children to make an individual decision (while the recipient was absent and could not influence the outcome).

Interestingly, a third of the children in Experiment 1 shared more than half of the rewards with their partner if s/he had contributed more, indicating that the majority of young children may have found it difficult to give generously. Previous studies have shown this behavior in children until nine years of age [9], which has been attributed to younger children systematically overestimating their own inferior work-efforts [7]. However, we ensured that children always correctly recalled the number of coins each partner had collected, ruling out the possibility that children systematically overestimated their own contribution. Alternatively, it is possible that children would share more generously with an actual child than with the puppet-partner that was used for purposes of experimental standardization. While this remains a possibility to be investigated in future studies, it is very likely that a similar self-serving bias remains when young children interact with other children. In fact, three-year-olds will react negatively to inequitable distributions when they receive less than a partner [17], [18], but it is not until eight years of age that they will actively prevent advantageous inequity, i.e. prefer receiving nothing over receiving more than a partner (e.g., [17]). Thus, the ability to apply merit in situations in which the outcome is disadvantageous to children probably does not develop until middle childhood. Future studies should investigate this possibility further by studying the merit principle in older children.

Finally, our study shows that young children can use comparisons between work-contribution to allocate resources. In our study, children could use either a number matching strategy (i.e. matching the number of rewards to the number of coins) or an ordinal scaling strategy, which may represent the most basic and developmentally early form of equity [4]. Future research should investigate at what age children begin to give exactly proportionally to work-contribution, including situations where an exact number-match of work-contribution and rewards is not possible (e.g. working towards collecting 100 items to share 4 rewards). It is unlikely, however, that proportional work-contributions would be considered at an early age, given the limited proportional reasoning skills in children younger than four years of age [19], [20].
