# Correction: Young Children Consider Merit when Sharing Resources with Others

**DOI:** 10.1371/annotation/221e5f19-370e-4a52-add8-f882437bc85d

**Published:** 2013-08-13

**Authors:** Patricia Kanngiesser, Felix Warneken

During the typesetting of the article, several typographical errors were introduced that were not present in the original manuscript. All errors concern missing decimal points in the Results section of the article. PLoS ONE takes full responsibility for these errors.

On page 2 (Data coding and analyses) F-statistic should read *F*_1,32_ = .71, *p* = .405, *η*_p_^2^ = .022. On page 2 and 3 (Results and discussion) F-statistics should read *F*_1,34_ = 16.32, *p* < .001, *η*_p_^2^ = .324; *F*_1,34_ = .47, *p* = .496, *η*_p_^2^ = .014; *F*_1,34_ = .10, *p* = .758, *η*_p_^2^ = .003; t-statistics should read *t*(35) = 2.77, *p* = .009, *d* = 0.94; *t*(35) = 0.41, *p* = .686, *d* = 0.14; and Chi-square statistic should read *χ^2^*(1, *N* = 36) = 0.11, *p* > .999. On page 4 (Data coding and analyses) F-statistic should read *F*_1,32_ =.49, *p* = .488, *η*_p_^2^ = .015. On page 4 (Results and discussion) F-statistic should read *F*_1,32_ = .24, *p* = .631, *η*_p_^2^ = .007; *F*_1,32_ = 7.93, *p* = .008, *η*_p_^2^ = .199; *F*_1,32_ = 7.00, *p* = .013, *η*_p_^2^ = .180; *F*_1,32_ = 7.00, *p* = .013, *η*_p_^2^ = .180; and t-statistics should read: *t*(8) = 1.35, *p* = .214, *d* = 0.96; *t*(8) = 1.89, *p* = .095, *d* = 1.34; *t*(8) = 1.41, *p* = .195, *d* = 1.00; *t*(8) = 1.00, *p* = .347, *d* = 0.71; *t*(8) = 1.35, *p* = .214, *d* = 0.96; *t_s_*(8) = 5.12, *p_s_* = .001, *d_s_* = 3.62.

